# Steatosis and Interferon Associated with HBsAg Immune Control in Chronic Hepatitis B: A Real-World Propensity Score-Matched Study

**DOI:** 10.3390/biomedicines13071538

**Published:** 2025-06-24

**Authors:** Qi Xu, Junjie Chen, Bilian Yao, Xinxin Zhang, Yue Han

**Affiliations:** 1 Department of Infectious Diseases, Research Laboratory of Clinical Virology, Ruijin Hospital, Shanghai Jiao Tong University School of Medicine, Shanghai 200025, China; xuqi1019@126.com (Q.X.); cjj8k6@163.com (J.C.); 2 Sino-French Research Centre for Life Sciences and Genomics, Ruijin Hospital, Shanghai Jiao Tong University School of Medicine, Shanghai 200025, China; 3 Department of General Practice, Ruijin Hospital, Shanghai Jiao Tong University School of Medicine, Shanghai 200025, China; ybl11578@rjh.com.cn; 4 Clinical Research Center, Ruijin Hospital, Shanghai Jiao Tong University School of Medicine, Shanghai 200025, China

**Keywords:** functional cure, HBsAg seroconversion, interferon, steatosis, propensity score matching

## Abstract

**Background/Objectives:** The baseline determinants of functional cure in chronic hepatitis B (CHB) are largely unknown. By applying propensity score matching (PSM) to real-world data, we aimed to identify traits associated with functional cure. **Methods:** We included CHB cases which achieved a functional cure and randomly selected non-achievers from patients followed from 2000 to 2020. Initial screening of baseline candidate traits was conducted using PSM-balanced cases and controls. Subsequently, through multiple rounds of leave-one-covariate-out on the balanced cohorts, we validated the impact of these traits using survival analysis. **Results:** In total, 85 cases (mean age: 35.78; female/male: 23/62) were compared with 247 controls (mean age: 37.08; female/male: 80/167, out of 3666), with a median follow-up of 69.56 months. Steatosis and interferon (IFN) treatment were significantly more frequent in the cases, as confirmed by forest plots showing significant hazard ratios. During validation, whether through balancing all covariates or leave-one-covariate-out matching, both steatosis and exposure to IFN resulted in a higher number of functional cures and HBsAg seroconversions. Further comparisons revealed that add-on or monotherapy outperformed switching (from IFN to NUC), while the de novo (IFN + NUC, followed by NUC) approach was not observed. **Conclusions:** We confirmed that individuals with steatosis at baseline or those who received IFN were more likely to achieve HBsAg immune control, with monotherapy/add-on therapy being emphasized.

## 1. Introduction

A functional cure [1] is defined by sustained hepatitis B surface antigen (HBsAg) loss along with undetectable HBV DNA six months after treatment cessation. It is associated with a higher remission rate and a reduced risk of cirrhosis and hepatocellular carcinoma. Unfortunately, it is uncommon and challenging to predict. Beyond this, HBsAg seroconversion to its neutralizing antibody (anti-HBs) represents a superior treatment outcome and is even rarer. A study has shown that liver-related mortality or events are similar regardless of HBsAg loss [2], suggesting that immune homeostasis may remain suboptimal without HBsAg seroconversion. No study has identified predictors of HBsAg seroconversion.

Indeed, numerous studies have associated low levels of hepatitis B virus (HBV) DNA, quantitative HBsAg, HBeAg negativity, gender, age, ALT, regimens incorporating immune modulators [3,4,5], and various combination strategies [6,7,8,9] with HBsAg loss. However, these studies often produce conflicting results [3,10,11,12], and have limited predictive value [13]. Recent evidence also highlights the role of steatosis in spontaneous HBsAg loss among treatment-naïve patients [11,14]. Whether this association extends to treated patients remains unclear.

To investigate the factors linked to HBsAg control under antivirals, two approaches can be employed: randomized controlled trials (RCTs) or real-world studies. It is important to recognize that RCTs, due to their high costs, are typically funded by the pharmaceutical industry. Therefore, selecting rare events as primary endpoints is not advisable, such as HBsAg loss or the even rarer HBsAg seroconversion. In drug RCTs, the focus is on the pharmaceutical molecule rather than host or viral traits. Real-world studies, while valuable, also have limitations, as they require a sufficient number of cases and a lengthy follow-up period. The heterogeneity observed in studies predicting HBsAg loss further underscores the multifactorial nature of this phenomenon [15]. Unsupervised real-world descriptive studies, often analyzed using multivariate methods, are inherently biased and confounded. Consequently, when using real-world data, it is essential to handle confounders with extra caution and interpret the results appropriately.

In this study, to optimize the use of real-world data, we conducted a thorough evaluation of potential covariates and employed a variety of debiasing strategies, including randomization and multilevel covariate matching, during both the screening and validation phases.

## 2. Materials and Methods

### 2.1. Definition of Case and Control

As part of a real-world long-term follow-up study of the Research Laboratory of Clinical Virology from January 2000 to December 2020, records of all subjects chronically infected with HBV for over 6 months and subsequently treated with antivirals were reviewed. Functional cure was defined as sustained HBsAg loss in addition to undetectable HBV DNA 6 months after treatment completion within the specified time frame. To minimize selection bias, we randomly selected controls from those who did not achieve a functional cure using the RAND() function provided by Microsoft Excel (Microsoft, WA, USA). Patients with co-infections, alcohol abuse, chronic liver disease, autoimmune diseases, or etiologies other than HBV were excluded. Elite controllers were defined as individuals who achieved HBsAg seroconversion following antiviral therapy. The Ethics Committee of Ruijin Hospital approved this study (201617/201848), with the protocol adhering to the principles of the Declaration of Helsinki and Good Clinical Practice.

### 2.2. Pre-Matching Statistics–Hypothesis Forming

To ensure fair comparisons between cases and controls, we must first identify the relevant covariates. In addition to HBsAg being a significant covariate affecting the outcome, we cannot exclude the possibility of other bias-inducing covariates. Factors such as gender, age at antiviral initiation, baseline transaminase (ALT) level, serum viral markers (viral loads, HBsAg quantifications, HBeAg status), antiviral regimens, and treatment duration (time to functional cure in cases of HBsAg loss) were compared between functionally cured cases and controls. The skewness of each variable was checked and plotted. Comparisons were made using parametric or non-parametric methods as appropriate. A *t*-test was used to evaluate continuous variables, such as age, HBeAg titers, HBsAg quantifications, viral loads, ALT levels, and treatment duration. Chi-squared tests were used for categorical variables, including sex, HBeAg status, regimens, combination strategies, and the presence or absence of steatosis. In parallel, we conducted a blind collinearity check to identify potential inter-related factors. Additionally, we created a pre-matching forest plot that included hazard ratios (HR) and significance levels. We incorporated these potential outcome/impact factors as propensity balance covariates, either because they aligned with previous research or demonstrated significant collinearity.

### 2.3. Propensity Score Matching (PSM)

Rounds of PSM were achieved using the R package ‘MatchIt’ (version 4.5.5). To obtain more balanced groups for comparisons, logistic regression modeled the probabilities of receiving the variable of interest, given the sets of covariates. After the initial estimation, a propensity score was calculated for each participant, reflecting the subject’s predicted probability of receiving the intervention of interest (i.e., being treated), based on the prior modeling estimates. The region of common support was examined using these scores. Nearest neighbor joining was then chosen to pair cases and controls with a matching ratio of 1. Following the completion of the matching, a series of balance checking methods were employed, including both tabular and graphical comparisons. Specifically, empirical quantile–quantile (Q-Q), cumulative distribution function (CDF), density, jitter, and histogram plots were generated. Standardized mean differences were computed to assess comparability of covariates. Once balance was achieved, outcome analysis was conducted using independent Welch Two Sample t-tests for continuous variables and chi-squared tests for categorical variables.

### 2.4. Candidate HBsAg Loss-Associated Factor Screening and Validation

For case-specific trait screening, to compare each variable listed in Table 1 more objectively, each of them was considered as the dependent variable, and t-tests or chi-squared tests were conducted between HBsAg loss cases and controls, provided that all other covariates were balanced through PSM. Subsequently, at the validation cohort stage, each candidate was considered the independent variable of interest, while other variables, except for treatment outcome, were balanced to form new exposed/non-exposed pairs for validation.

### 2.5. Survival Analysis

Cumulative event curves, stratified by the to-be-validated candidates, were plotted, and hazard ratios were also calculated.

### 2.6. Leave-One-Covariate-Out Analysis

**Figure 1:**
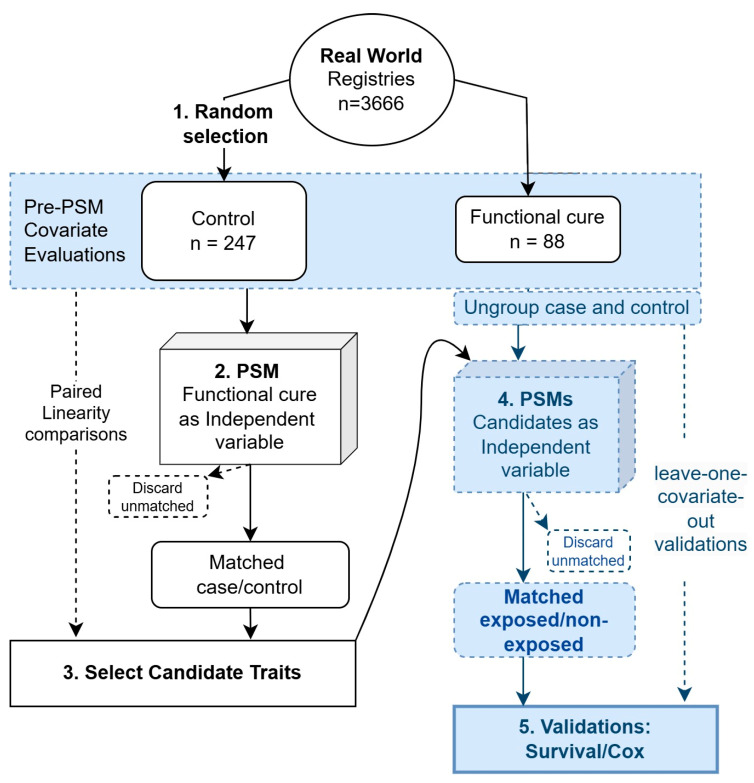
Schematic view of the analytic flow. The analytic procedures were numbered sequentially from 1 to 5.

To make the comparisons fairer and also for validation purposes, while taking into account the possible intrinsic relationships between the selected variables, we adopted the leave-one-covariate-out strategy to ensure a thorough analysis. Specifically, to evaluate the impact of candidate traits on the accumulation events of either HBsAg seroclearance or seroconversion, we excluded one covariate at each step to check the consistency of their roles in the outcome. Covariates were selected based on paired linearity comparison results, as these covariates might influence the outcome in a more complex manner. All the aforementioned analyses were conducted using R (version 4.0.5) with the basic and mentioned packages. Figure 1 shows the analytic flow.

## 3. Results

### 3.1. The Proportions of Steatosis Cases and Interferon (IFN) Usage Differed Significantly Between the Cases and Controls, Regardless of PSM

From a 20-year follow-up database of 3666 patients, we retrospectively analyzed 85 cases of functional cure and randomly selected 247 controls. Age, sex, HBeAg status, HBeAg semi-quantifications, viral loads, ALT, and treatment duration were comparable between the functionally cured cases and the control group. A lower baseline HBsAg level was observed in the cured cases, as expected. Additionally, a higher prevalence of steatosis diagnoses and a more frequent use of IFN-containing regimens were noted in the cured cases. To further compare IFN inclusion and steatosis between cases and controls, we balanced all the aforementioned confounding factors. Detailed descriptions of the significance value before and after balancing, along with support from the literature, are provided in Table 1. Both IFN and steatosis remained significantly different after adjusting for covariates.

### 3.2. Paired Linearity Checks for Potential Inter-Covariate Interferences

We considered there to be no relationship between covariates when the r value was less than 0.25, a weak relationship when the value was between 0.25 and 0.5, a moderate relationship when 0.5 < r < 0.75, and a strong relationship when r > 0.75. Among all included patients, we found weak correlations between HBsAg levels and HBeAg semi-quantifications (positive correlation, r = 0.414), as well as between age and HBeAg (negative correlation, r = −0.380). Younger age was associated with IFN inclusion (r = −0.321). Among the cases, the aforementioned weak linear relationships still existed between HBsAg levels and HBeAg semi-quantifications (r = 0.428), age and HBeAg (negative correlation, r = −0.265), and age and IFN inclusion (r = −0.426). Among controls, weak linear relationships were observed between HBsAg levels and HBeAg semi-quantifications (r = 0.421), age and HBeAg (negative correlation, r = −0.422), and age and IFN inclusion (r = −0.290). Additionally, HBV viral load exhibited a positive correlation with both HBeAg (r = 0.399) and HBsAg (r = 0.610). Thus, no strong relationships were found between the covariates. The only moderate relationship identified was between viral load and HBeAg in the control group. ALT did not exhibit any significant linear associations. Therefore, based on the observed significance level and in line with the literature, in the subsequent PSM-matched process, we included HBsAg levels, HBeAg, age, IFN inclusion, treatment duration, and viral load, but excluded ALT, as potential covariates.

### 3.3. The HR of IFN Remained Significant for Functional Cure After Steatosis Ratio Balanced

**Figure 2:**
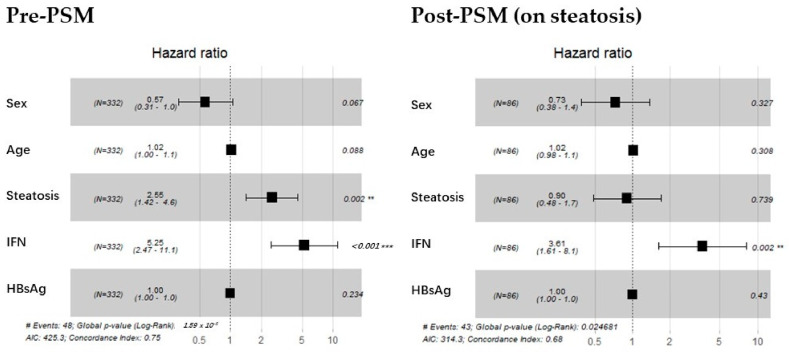
Screening stage: forest plot for Cox proportional hazards model before and after debiasing on steatosis. The (**left**) graph displays the hazard ratios for sex, age, steatosis, IFN, and baseline HBsAg level before matching. The (**right**) graph presents the same analysis, with steatosis treated as a strong covariate and balanced. Dotted vertical line represents a ratio of 1. The position of a black square is a point estimate of the ratio. **: *p* < 0.01 (highly significant); ***: *p* < 0.001 (extremely significant).

The pre-PSM forest plot in Figure 2 indicates that the HR for steatosis and IFN inclusion was 2.55 and 5.25, respectively, before matching. Regardless of steatosis bias, the HR of IFN continued to be significant for functional cure, as shown in the post-PSM section of Figure 2. Interestingly, once the IFN user proportion was debiased, steatosis no longer exhibited a significant hazard. This suggests a potential synergistic effect between the two factors or an overlap in their demographic characteristics.

### 3.4. Steatosis and IFN-Based Regimens Confirmed as Being Associated with Functional Cure and HBsAg Seroconversion During the Validation Stage

**Figure 3:**
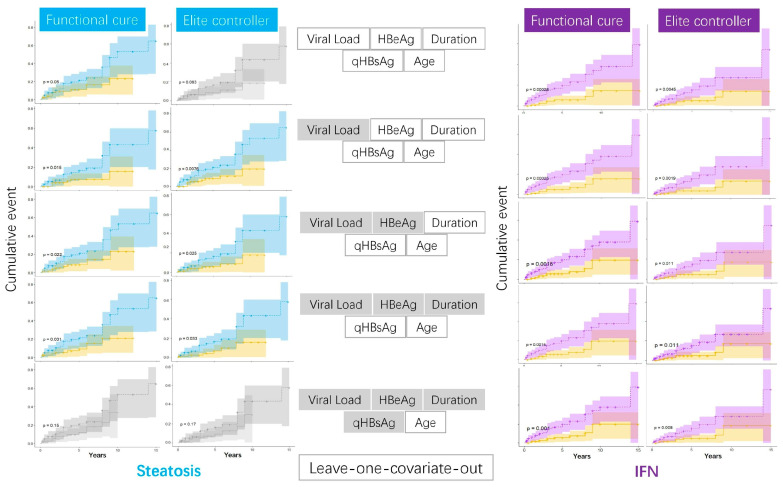
Validation stage: exhaustive leave-one-covariate-out analysis of steatosis/IFN on HBsAg outcomes. The left blue plots show survival analysis based on steatosis status, while the right purple plots represent survival outcomes depending on whether IFN was used. Plots with significant levels are highlighted in color. The middle section indicates the covariates being considered, with grayed-out covariates being excluded.

To validate the role of steatosis or IFN inclusion in achieving either a functional cure or HBsAg seroconversion, we conducted extensive validations using leave-one-covariate-out balancing in cumulative events analysis. IFN remained consistently significant. Steatosis also maintained its significance in most scenarios, except when age was the covariate being balanced, as shown in Figure 3. For exploratory purposes, we also plotted the age distribution among IFN users, stratified by HBeAg status and steatosis, with unbalanced cases and controls. We demonstrated that HBeAg-positive patients with early-onset steatosis might benefit more from IFN treatment (in-house data).

### 3.5. IFN Regimens Varied, with Add-On and Monotherapy Being Preferred

**Figure 4:**
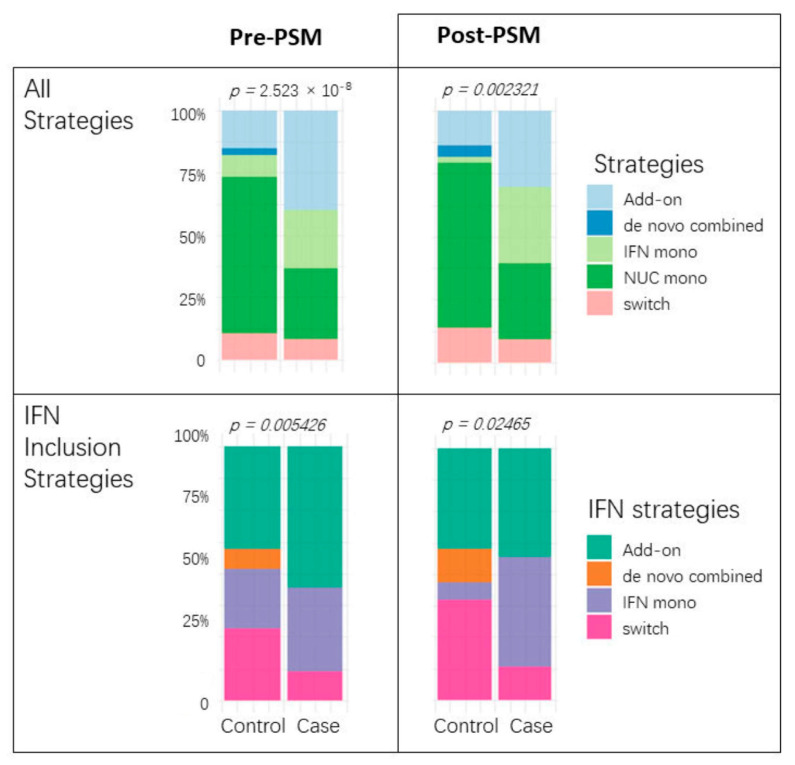
Antiviral strategy comparisons before and after PSM. Each paired bar plot illustrates the proportion of treatment choices between cases and controls. The (**upper**) two graphs encompass all choices, including NUC monotherapy, while the (**lower**) two focus solely on comparing IFN strategies.

Having clarified the role of IFN, we further examined the differences in treatment responses among four types of IFN inclusion regimens: add-on (NUC therapy followed by the addition of IFN), de novo combined (initial concurrent use of IFN and NUC), IFN monotherapy, and switch (transition between IFN and NUC). The switching regimen, which involves the sequential use of the two drugs without overlap and is mostly IFN followed by NUC, was less common, and no de novo cases (IFN + NUC followed by NUC) were observed. Regardless of debiasing, NUC monotherapy was less frequently seen in cases, which aligns with the emphasis on IFN therapy. Additionally, irrespective of covariate balancing, no cured cases had chosen the de novo combined strategy. The majority of cases using IFN had opted for the add-on or IFN monotherapy approaches. Switching to IFN represented a very small proportion of cured cases, as illustrated in Figure 4.

## 4. Discussion

This study is the first to establish a connection between steatosis and both functional cure and HBsAg seroconversion, a more favorable outcome, through the application of an extensive debiasing process to a real-world dataset. The PSM algorithm created a quasi-experimental environment to assess the impact of any variable of interest on the outcome while avoiding propagating biases. Additionally, we confirmed that interferon (IFN) enhances immune control over surface antigen, with combination or monotherapy proving more effective than a de novo approach.

At the screening stage, baseline HBsAg level [3,10], IFN-included regimen [3], treatment duration, and steatosis [11] were identified as being associated with outcome, consistent with previous reports. In contrast, factors such as age [3,17], gender [3,16,18], HBeAg-negative status [3,10], viral loads [10,16], and ALT levels [19], which have yielded conflicting results in prior studies, did not show significant differences between cases and controls. This high replicability strongly supports the chosen methodology.

HBsAg levels are undoubtedly a focal point of current research interest [22,23]. However, the limitation of their use is also evident. Using HBsAg levels to predict HBsAg loss renders the independent and dependent variables identical, which is mathematically meaningless and excludes many patients who could potentially benefit from treatment. At the 2024 American Association for the Study of Liver Diseases’ annual meeting, Kosh Agarwal’s group demonstrated that HBeAg-positive patients with high baseline HBsAg levels experienced higher rates of HBsAg loss (abstract title: “Kinetics of Hepatitis B Surface Antigen Loss Following 8 Years of Tenofovir-Based Treatment in HBeAg-Negative and HBeAg-Positive Patients with Chronic Hepatitis B”). Therefore, it is more reasonable to consider HBsAg levels as a covariate to balance in the search for other predictive factors, rather than basing decisions primarily or exclusively on this metric.

The confirmation of the role of IFN therapy holds significant implications both clinically and methodologically. According to the largest meta-analysis [10] to date, the annual rate of HBsAg loss [24] remains comparable [3] between treated and naïve cases, seroclearance occurs primarily in patients with less active disease (community-based compared to hospital/clinic-based cohorts), regardless of treatment. This implies that the current treatment strategies, on a macro level, are not optimal. Indeed, NUCs can reduce the antigen burden: evidence from the 2017 T. Berg study shows that Tenofovir achieved a 19% clearance rate [25]. However, Siederdissen HZ reported that virological and clinical relapse was seen earlier with Tenofovir compared to Entecavir [26], suggesting that even the most potent NUC, when used alone, is insufficient for immunological control. HBsAg contributes to the dysregulation of both innate and adaptive immune cells [12]. HBV deploys tens of thousands of surface antigens to exhaust and disrupt host immunity [27]. While functionally cured patients showed reactivation of innate and adaptive CD4-driven immunity [28], evidence also showed that simple HBsAg loss does not trigger effective T cell responses [29]. Recently, in cancer patients undergoing immune checkpoint inhibitor therapy with very low baseline HBsAg levels, an acceleration of HBsAg seroclearance was noticed [5], emphasizing the importance of introducing immune modulators. Regarding IFN inclusion strategies, the approach of using NUC and IFN together from the start of treatment appears ineffective in achieving HBsAg loss, suggesting that timing of introducing IFN is a critical factor. A recent systematic review also concluded that the efficacy of the de novo combination was not superior to that of PEG-IFNα monotherapy in terms of functional cure [30]. We demonstrated that the inclusion of IFN is consistently superior to NUC alone in achieving both HBsAg loss and seroconversion. This underscores the robustness of our debiasing approach, again.

Most intriguingly, the importance of steatosis has been revealed. Recent evidence suggests steatosis plays a role in spontaneous HBsAg loss among naïve patients [11,14], which aligns well with our findings in treated cases. Steatosis has the potential to evolve into metabolic dysfunction-associated liver disease, the pathophysiology of which involves innate and adaptive immune responses [31]. An overlap between steatosis and HBV immune response might exist. Studies on bacterial infections using fatty liver mouse models have shown a trade-off between immunity and metabolism under infection and dietary pressure [32]. However, in this study, lacking a plausible explanation, the significance was lost when age was the only factor balanced. This suggests the following: (1) steatosis’s unexplained role is indirect compared to IFN, (2) given that the progression of steatosis takes time, both steatosis and age should be considered in treatment decisions. This is partially evidenced by our exploratory density plots of the age distribution of HBeAg-positive IFN users (in-house data), which show that patients with early-onset steatosis might benefit more from IFN treatment. The exact mechanism by which steatosis influences viral activity remains to be elucidated through controlled viral host studies.

## 5. Conclusions

In this real-world propensity score-matched screening and validation study, we confirmed that both IFN and steatosis promote the occurrence of HBsAg clearance, and even the ultimate immune control of HBsAg—the seroconversion. Specifically, regarding the use of IFN, monotherapy or add-on is more commonly observed among HBsAg loss achievers. Steatosis, as a baseline host trait, has the potential to serve as an independent predictor.

## Figures and Tables

**Table 1:** Baseline demographics of functional cure cases and controls.

	Functional Cure n = 85	Control n = 247	Sig. (2-Tailed) Before Matching	Balanced *	Literature Support
Age, Mean ± SD	35.78 ± 12.046	37.08 ± 12.247	0.394		older [3,16], younger [17]
Sex F/M	23/62	80/167	0.435		male [1,11], female [18]
HBeAg positive/negative	54/31	163/84	0.694		Negativity [3,10]
qHBeAg, Mean ± SD	640.77 ± 603.97	526.80 ± 713.53	0.226		NA
Viral load, Mean	2.11 × 10^8^	3.68 × 10^7^	0.247		lower load [10,16]
qHBsAg, Mean ± SD	10,131.79 ± 16,858.33	17,227.94 ± 25,971.53	0.022		lower level [3,10]
ALT, Mean ± SD	167.32 ± 298.41	123.70 ± 154.33	0.246		higher level [19]
Treatment duration, Mean± SD	5.46 ± 4.41	5.91 ± 3.79	0.395		NA
Regime NUC/IFN/Combined	24/19/42	156/18/73	0.000		Yes
IFN included regime or not	61/24	91/156	1.181 × 10^−8^	0.01692 †	Discussed IFN benefits [20]
Combined strategy: add-on/switch/other	35/7/0	38/26/7	0.000		Favored switch over NUC alone [21]
Steatosis/non-steatosis	41/44	68/179	0.000	0.04722 ‡	Evidence from naïve cases

* Outcome differences before and after covariate balancing. Pearson’s chi-squared test of IFN, steatosis post age/sex/qS covariate balancing. † (qHBsAg, HBeAg, age, steatosis, duration, viral load as covariates) to judge IFN differences. ‡ (qHBsAg, HBeAg, age, IFN, duration, viral load as covariates) to judge steatosis differences.

## Data Availability

Data are unavailable due to privacy or ethical restrictions.

## Abbreviations

The following abbreviations are used in this manuscript:

CHB	Chronic hepatitis B
PSM	Propensity score matching
IFN	Interferon
NUC	Nucleoside/nucleotide analog
